# Torsional and Bending Properties of V Taper 2H, ProTaper NEXT, NRT, and One Shape

**DOI:** 10.1155/2019/6368958

**Published:** 2019-11-07

**Authors:** Soram Oh, Kee-Yeon Kum, Kwon Cho, Soo-Hyuk Lee, Seung-Hyun You, Jonggun Go, Bong-Ki Jeon, Sang-Woo Kim, Tae-Hwan Kim, Ji-Hyun Jang, Hiran Perinpanayagam, Jin-Woo Kim, Seok Woo Chang

**Affiliations:** ^1^Department of Conservative Dentistry, KyungHee University Dental Hospital, 23 Kyungheedaero, Dongdaemun-gu, Seoul 02447, Republic of Korea; ^2^Department of Conservative Dentistry, Dental Research Institute, Seoul National University Dental Hospital, School of Dentistry, Seoul National University, 101 Daehak-ro, Jongno-gu, Seoul 03080, Republic of Korea; ^3^The First Dental Clinic, 14 Siga-ro, Deoksan-eup, Jincheon-gun, Chungcheongbuk-do 27875, Republic of Korea; ^4^Department of Conservative Dentistry, Graduate School, Kyung Hee University School of Dentistry, 23 Kyungheedaero, Dongdaemun-gu, Seoul 02447, Republic of Korea; ^5^Department of Conservative Dentistry, Kyung Hee University School of Dentistry, 23 Kyungheedaero, Dongdaemun-gu, Seoul 02447, Republic of Korea; ^6^Schulich School of Medicine & Dentistry, University of Western Ontario, 1151 Richmond Street, London ON N6A3K7, Canada; ^7^Department of Conservative Dentistry, College of Dentistry, Gangneung-Wonju National University, 7 Jukheon-Gil, Gangneung, Kangwon-do 25457, Republic of Korea

## Abstract

Nickel-titanium (NiTi) rotary files have enabled efficient root canal preparations that maintain the canal center with fewer aberrations compared to hand files. However, NiTi rotary files are susceptible to fracture, which can thereby compromise root canal treatment. Therefore, NiTi files have been developed to enhance fracture resistance by modifying design and thermal treatment. The objective of this study was to compare the torsional fatigue resistance and bending resistance of NiTi files manufactured from different alloys and treatments. ProTaper NEXT X2 (PTN; M-wire), V taper 2H (V2H; controlled memory wire), NRT (heat-treated), and One Shape (OS; conventional alloy) instruments of tip size #25 were compared. Torsional fatigue was evaluated by embedding the 3 mm tip of each instrument (*N* = 10/brand) in resin and the repetitive application of torsional stress (300 rpm, 1.0 N·cm) by an endodontic motor with autostop when the file fractured. The number of loading cycles to fracture was recorded and analyzed by Kruskal–Wallis and Mann–Whitney *U* tests with Bonferroni's correction. Bending resistance of the instruments was tested using a cantilever bending test to the 3 mm point from the tip (*N* = 10/brand). The stress was measured when deflection of 3 mm was subjected and statistically analyzed with a one-way analysis of variance and Tukey's honest significance difference test (*α* = 0.05). V2H withstood the highest number of load applications during torsional fatigue testing (*p* < 0.05), followed by NRT, PTN, and OS, where the differences between NRT and PTN (*p*=0.035) and between PTN and OS (*p*=0.143) were not statistically significant. V2H showed the lowest bending stiffness, followed by NRT, PTN, and OS (*p* < 0.001). Thermal treatment of NiTi wire resulted in improved mechanical properties, and controlled memory wire provided improved flexibility and torsional fatigue resistance.

## 1. Introduction

NiTi rotary instruments have enabled easier and faster root canal preparations that maintain the canal center with fewer aberrations [1]. However, NiTi rotary files may separate during instrumentation [2], which then disrupts canal preparation and disinfection [3]. These file fractures can be attributed to torsional and cyclic fatigue [2]. Cyclic fatigue fracture occurs when a NiTi file is subjected to repeated cycles of tension and compression in a curved canal [4]. Torsional fracture occurs when the tip or portion becomes wedged in the canal, and the shaft continues to rotate [4]. To avoid fracture, various geometric and dimensional modifications have been incorporated into instrument designs. These strategies have included surface treatment such as electropolishing, modified cross section that reduce instrument contact with root canal wall, and a variable taper rather than constant taper in instrument design [5, 6].

NiTi alloy exists in two different temperature-dependent structures, namely, austenite (high-temperature or parent phase) and martensite phase (low-temperature phase) [7]. Martensite is soft and ductile and can be easily deformed than austenite. Under certain conditions, an R-phase may appear during temperature-dependent phase transformation between austenite and martensite. The elastic modulus of martensite is lower than that of austenite, and that of R-phase is lower than that of martensite [7]. Recently, thermal treatment of the NiTi wires such as R-phase wire (SybronEndo, Orange, CA, USA), M-wire, and controlled memory (CM) wire have been used in the manufacturing process to enhance their mechanical properties [8, 9]. While conventional NiTi files, which were not subjected to specific thermal treatment, consist of austenite at room temperature, NiTi files manufactured by thermal treatment possess certain amount of martensite or R-phase structure at room temperature [7, 9].

M-wire is a superelastic NiTi wire that contains some R-phase and martensite under clinical condition [9]. ProTaper NEXT (PTN; Dentsply Maillefer, Ballaigues, Switzerland), made from M-wire, exhibited superior cyclic fatigue resistance compared to NiTi files made from conventional alloy [10]. M-wire NiTi file contains deformed and microtwinned martensite, which accounts for higher tensile strength than conventional NiTi alloy [9]. Additionally, ProTaper NEXT showed greater torsional resistance compared to conventional or other heat-treated NiTi rotary files [11, 12].

CM wire is manufactured by a thermomechanical process that modifies its phase transition temperature [8], to attain superior flexibility and resistance to cyclic fatigue [13, 14]. However, CM wire does not possess the superelasticity of conventional NiTi, and superior torsional resistance has not been demonstrated for CM wire. A previous study reported that a file made from CM wire exhibited greater torsional resistance than an R-phase wire file (Twisted File; SybronEndo) [15]. Conversely, another study found that the maximum torque at failure of HyFlex CM (Colténe/Whaledent, Inc, Cuyahoga Falls, OH, USA) file, made from CM wire, was lower than those of GT series X and ProFile Vortex (Dentsply Tulsa Dental Specialties, Tulsa, OK, USA) that were made from M-wire [16]. V taper 2H (V2H; SS White, Lakewood, NJ, USA), manufactured using CM wire, presented superior flexibility and resistance to cyclic fatigue fracture than V taper 2 (SS White) which is made from conventional NiTi alloy [17]. However, the maximum torque of V taper 2 and V taper 2H was comparable [17].

NRT (Mani, Tochigi, Japan) is manufactured by the thermal treatment, and according to the manufacturer, only 5 mm tip is subjected to the thermal treatment. Superior cyclic fatigue resistance of NRT has been observed when compared to other NiTi files [18]. The manufacturer claims that the modified rectangular cross section strengthens its core. One Shape (OS; Micro Mega, Besançon, France), made of a conventional NiTi alloy, is a single-file system that rotates continuously. The manufacturers claim that their asymmetrical cross section reduces the risk of the instrument separating from accumulated strain on the file.

The measurement of torsional strength in NiTi instruments has been performed with a torsiometer, according to the International Standards Organization (ISO) 3630-1. The file tip is clamped rigidly and a torsional moment applied. At the time of fracture, maximum torque and angular deflection are recorded. However, these static conditions rarely occur during torsional failures in clinical practice. Therefore, Park et al. have developed torsional fatigue testing with a torque-controlled endodontic motor to better mimic clinical conditions [19].

The objective of this study was to compare the torsional fatigue resistance of PTN, V2H, NRT, and OS by using a torque-controlled endodontic motor that simulated their use in clinical practice. Additionally, their flexibility was compared by measuring the bending resistance of these NiTi rotary files. The null hypothesis was that there were no differences in torsional fatigue resistance or bending resistance between the PTN, V2H, NRT, and OS instruments.

## 2. Materials and Methods

NiTi rotary files PTN (Dentsply Maillefer), V2H (Colténe/Whaledent, Inc), NRT (Mani), and OS (Micro Mega) of 21 mm length and identical ISO tip size #25 were selected. NRT and OS have a constant 6% taper. PTN has a 6% taper at 3 mm from the tip , followed by a 7% taper up to 9 mm, and then the taper decreased up to 16 mm. V2H has a 6% taper at the tip area, followed by a decreasing rate of taper along the shaft. OS and V2H have convex triangular-shaped cross sections at 3 mm from the tip, PTN has a rectangular cross section, and NRT has a modified rectangular cross section at 3 mm from the tip, according to the manufacturers.

**Figure 1:**
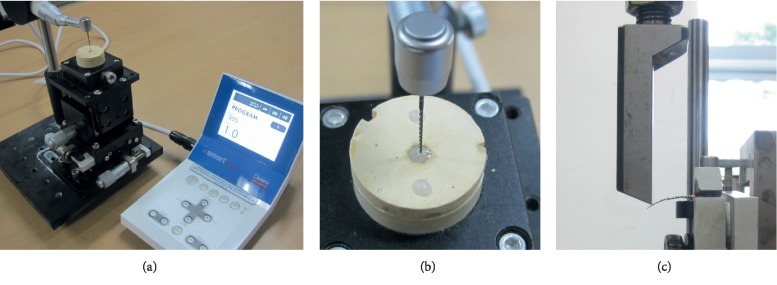
Test devices used in the present study. (a) Setup for torsional fatigue resistance test. (b) A NiTi rotary file was secured in a composite resin in which a torsional resistance test was performed. (c) Setup for cantilever bending test using a universal testing machine.

The torsional fatigue resistance of each instrument (*N* = 10/group) was evaluated according to the method described by Park et al. [19]. Torsional fatigue resistance was evaluated alone without the influence of flexural fatigue by the application of torsional stress to the file while it was straight. The tip (3 mm) of each file was inserted into a composite resin block (G-aenial Universal Flo, A2 shade; GC, Tokyo, Japan) and light-cured (Figure 1(a)). Then the file shaft was fastened into a chuck that was connected to a torque-controlled endodontic motor (X-smart, Dentsply Maillefer), with the maximum torque set at 1.0 N·cm (Figures 1(a) and 1(b)). Each file was rotated clockwise at 300 rpm until the preset torque was reached and the engine stopped automatically, which counted as one loading cycle. The motor was turned on again and the process repeated until the file fractured. For each file, the number of loading cycles to fracture was recorded and analyzed by Kruskal–Wallis and Mann–Whitney *U* tests with Bonferroni's correction. The fracture surfaces (*N* = 3/group) were cleaned, sputter-coated with a conductive carbon in a sputter coating unit (Q150TS: Quorum Technologies, Lewes, East Sussex, UK), and examined by scanning electron microscope (SEM; S-4700, Hitachi High Technologies, Pleasanton, CA, USA).

The bending resistance of each file (*N* = 10/group) was evaluated by a cantilever bending test, according to the previous study with a universal testing machine (Instron 5583, Instron Corp., Norwood, MA, USA) [20]. Each file handle was secured to a chuck in a perpendicular orientation to the axis of the geared motor (Figure 1(c)). Then a load was applied by a bending lever (blade) that was attached to the motor of the universal testing machine. Initially, when the bending lever was placed slightly above the file, a baseline (zero) was recorded by integrated computer software. Then, load was applied in a downward direction at a point 3 mm from the tip of each file (1.0 mm/min) until a vertical deflection of 5.0 mm was achieved. The bending resistance was measured with a stress to the bending lever when it moved 3.0 mm vertically.

Three unused instruments in each group were embedded in a clear resin and sectioned 3 mm from the tip with an IsoMet Low Speed Saw (Buehler, Lake Bluff, IL, USA). A SEM was used to examine the cross section, and then the cross-sectional area and inner core area at 3 mm cross sections were measured using ImageJ software (http://rsbweb.nih.gov/ij). Additionally, new instrument of each brand was examined to figure out helical angle and pitch length, and the helical angle was measured using ImageJ software.

The statistical difference in the bending resistance was analyzed with a one-way analysis of variance and Tukey's honest significance difference test (*α* = 0.05).

## 3. Results

Table 1 outlines the minimum, maximum, and mean numbers of torsional stress applications to fracture. V2H had the greatest resistance to torsional fatigue (*p* < 0.001) and OS the least. When two brands of NiTi files were compared with Mann–Whitney *U* test, there were 6 comparisons. After adjusting Bonferroni's correction, *p* value lower than 0.0083 (0.05/6) was regarded as statistically significant. The differences between OS and PTN (*p*=0.143) and the difference between PTN and NRT (*p*=0.035) were not statistically significant. The NRT was significantly resistant to torsional fatigue compared with OS (*p*=0.002).

**Figure 2:**
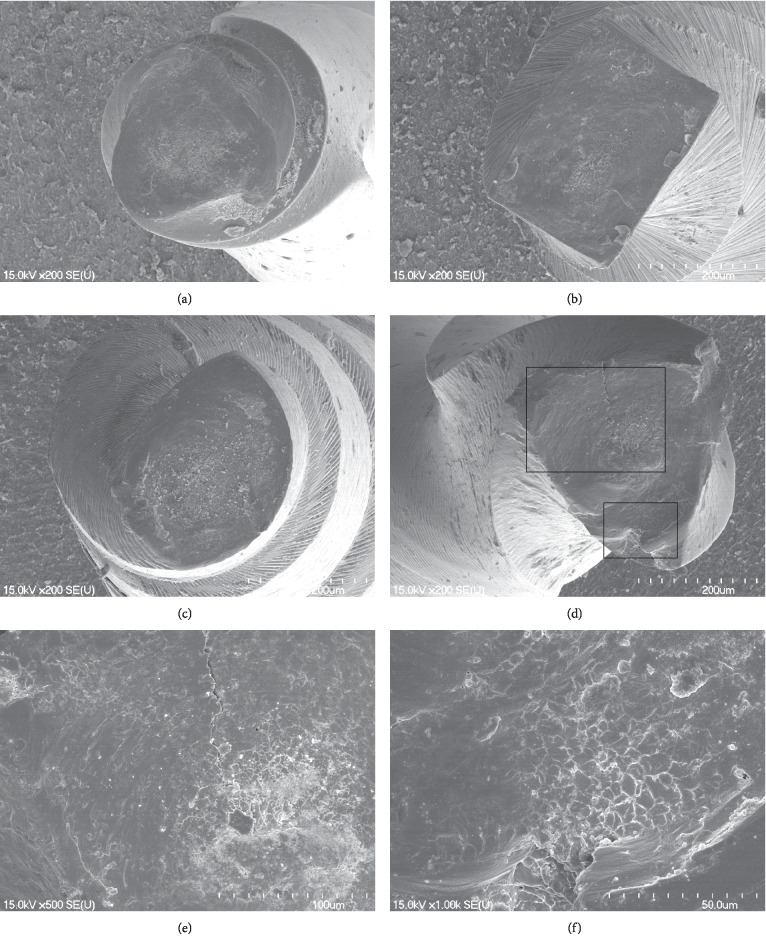
Scanning electron microscopy photographs of the fractured surface of NiTi files after the torsional fatigue resistance test. Fractured surfaces of the One Shape (a), ProTaper NEXT (b), NRT file (c), and V taper 2H (d) (original magnification, x200) showed circular abrasion marks and skewed dimples at the center of the rotation region. (e) Magnified view of upper box of (d) showing a crack line from the boundary to the central region (x500). (f) Magnified view of lower box of (d) showing circular abrasion marks at the periphery of the fractured surface (x1,000).

The fractured surfaces of NRT, OS, and PTN had a topography that showed typical patterns of torsional fracture with circular abrasion marks (Figures 2(a)–2(c)). However, the fractured surface of V2H had a cracked line that extended from the fracture boundary to the center of the file (Figures 2(d) and 2(e)). Furthermore, the fractured surface of V2H had a smaller area of circular abrasion marks than the fractured surfaces of other files (Figure 2(d)). On fractured V2H, there was a torn-off appearance, with a rough, uneven surface, and dimples at the periphery of one instrument, unlike the other files (Figures 2(d) and 2(f)).

**Figure 3:**
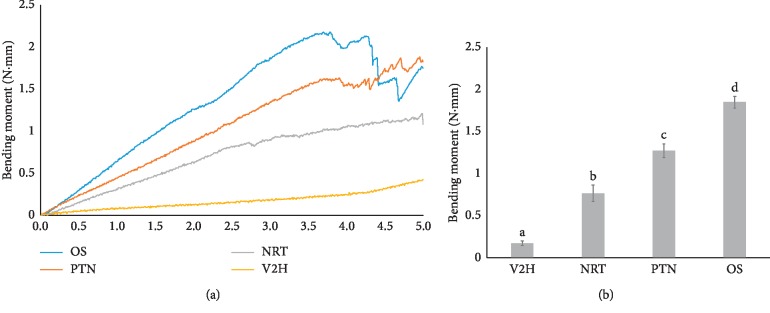
Results from the bending resistance test. (a) Representative graph of bending moment (N·mm)-deflection (mm) curves obtained in the bending resistance test. (b) Bending moment of four different NiTi rotary files. Groups with different letters indicate a statistically significant difference (*p* < 0.001). OS, One Shape; PTN, ProTaper NEXT; V2H, V taper 2H.

Bending resistance test result is presented in Figure 3. Deflections of 3.0 mm at 3 mm from the file tip were found to be within the range of elastic deformation for all instruments (Figure 3(a)). At a deflection of 3.0 mm, the bending resistance was lowest in V2H and increased progressively from NRT to PTN and OS, which had the highest. When the pairwise comparisons were performed using Tukey's honest significance difference test, differences from all comparisons were statistically significant (*p* < 0.001).

**Figure 4:**
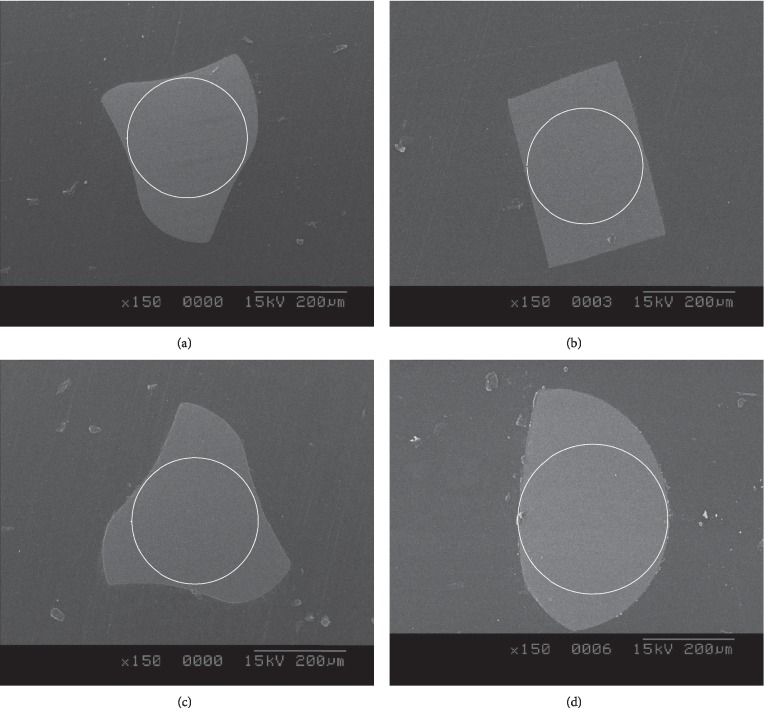
3 mm cross sections from tip of unused files. (a) One Shape; (b) ProTaper NEXT; (c) V taper 2H; (d) NRT. The inner white circle represents the central inner core for each instrument.

**Figure 5:**
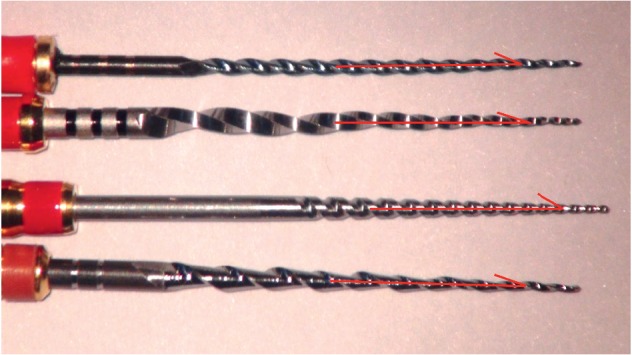
Lateral surfaces of unused instruments. The angle between two red lines is a helical angle of the NiTi file. From top to bottom, V taper 2H, ProTaper NEXT, NRT, and One Shape.

At 3 mm from the file tip, the cross sections of OS and V2H were convex triangular-shaped, whereas that of PTN was rectangular-shaped and that of NRT was teardrop-shaped (Figure 4). The cross-sectional area of NRT was the largest, followed by V2H, PTN, and OS, which was the smallest (Table 2). Additionally, the inner core area of NRT was the largest, followed by V2H, OS, and PTN, which was the smallest. Unused instrument of each brand is shown in Figure 5. The helical angle of V2H, PTN, NRT, and OS was 26.2°, 24.6°, 30.4°, and 18.3°, respectively.

## 4. Discussion

Several factors influence the torsional resistance of NiTi rotary files. These include size, taper, flute depth, pitch length, core area, cross-sectional shape, and alloy treatments [16, 20–24]. In the present study, the V2H manufactured from controlled memory wire exhibited superior torsional fatigue resistance to files from superelastic NiTi alloy. It is speculated that the proprietary thermal treatment of V2H may have annealed the material and reduced the residual internal stress produced in the mechanical process [17].

A large number of threads are associated with shorter pitch length and greater helical angle. Prior studies reported that NiTi rotary files that have shorter pitch length and greater helical angle exhibit superior torsional resistance [24, 25]. The number of threads for PTN, V2H, NRT, and OS are 7, 10, 9, and 7, respectively. Therefore, the large number of threads of V2H and NRT may have contributed to their higher torsional fatigue resistance. Both V2H and NRT demonstrated superior resistance to torsional fatigue as well as bending flexibility. This finding corroborated He et al., who reported that a greater helical angle offered both bending flexibility and torsional stiffness to the instrument [25].

Proprietary thermal treatments for the instruments were patented by the manufacturers and the details disclosed. It appears that M-wire possesses some martensite and R-phase, and NRT possesses some R-phase NiTi at room temperature [9, 26]. However, the major difference between NRT and PTN is in their cross-sectional geometry. The cross-sectional and inner core areas at 3 mm from the tip in NRT are much larger than in PTN and OS (Figure 4, Table 2). This geometric factor contributed to the higher torsional fatigue resistance of NRT compared to PTN and OS, although the difference between NRT and PTN was not statistically significant. While the manufacturer claimed the cross section of NRT was rectangle, the 3 mm point was teardrop-shaped based on the SEM image (Figure 4(d)) [27].

Fractographic analysis by SEM revealed circular abrasion marks and skewed dimples around the center of rotation of the fractured surfaces for OS, PTN, and NRT (Figures 2(a)–2(c)). In contrast, fewer circular abrasion marks were observed for V2H (Figure 2(d)), and a crack line was observed for one fractured specimen (Figures 2(d) and 2(e)) that was presumed to be a cause of the fracture. The CM wire is thought to be able to endure greater torsional stress prior to fracture due to a martensitic transformation. There is no recommended limit on V2H file usage, yet the appearance of file distortion may indicate the possibility of instrument fracture. Therefore, V2H files should be inspected following autoclave sterilization since NiTi files made from CM wire may largely return to their original shape after undergoing minor deformations [28].

Torsional fracture occurs when the instrument tip is wedged within constricted canal space and the shaft continues to rotate [29]. This taper lock effect that was utilized in this study closely reflects clinical conditions [19]. The torque-controlled motor and maximum torque values for the NiTi rotary instruments were provided as per manufacturer recommendations. The recommended maximum torque values are 2.5 N·cm for OS, 2.0 N·cm for PTN and V2H, and 2.45–2.94 N·cm for NRT. However, all OS instruments fractured following a single load application of 1.0 N·cm. These experimental conditions may have been extreme, but the OS is used as a single-file operating system and is therefore subjected to higher stresses during instrumentation.

To evaluate the bending properties of endodontic instruments, the ISO 3630-1 established a bending test, which involves clamping 3 mm of the tip of each instrument into a chuck and applying an angular deflection of 45° [30]. However, as the ISO test was established for stainless steel files, this study measured the bending moment for NiTi rotary files by using a cantilever bending test [20]. Bending moment-deflection curves showed that a deflection of 3 mm at 3 mm from the file tip was within the elastic deformation limit for all instruments.

The flexibility of these NiTi rotary files is depended on the properties of their alloy, rather than their cross-sectional geometry. OS, which was made from conventional NiTi alloy, exhibited the highest bending stiffness (*p* < 0.05), although it presented the smallest cross-sectional area among the tested NiTi files. This finding has corroborated with previous studies [31, 32]. The greater flexibility of heat-treated instruments is attributed to modification of their transformation temperature. The presence of martensite or R-phase NiTi at room temperature contributed to the enhanced flexibility of those instruments, due to lower Young's modulus compared to austenite state [8]. The critical stress to induce martensitic reorientation (twinned to deformed martensite) in martensitic NiTi file is much lower than the critical stress to cause stress-induced martensitic transformation (austenite to deformed martensite) in austenitic NiTi file [7].

V2H presented the lowest bending moment (*p* < 0.05). According to Chang et al., differential scanning calorimetry of V2H showed that it was composed of a mixture of austenite and R-phase at room temperature, since the austenite finish temperature (33.25°C) is above room temperature [17]. The critical plateau stress of CM wires has been reported to be much lower than that of the superelastic wires for stress-induced martensitic transformation [13]. However, the maximum strain in CM wire before fracture is higher than that in superelastic wire [13], so that CM wire exhibits more flexibility than superelastic NiTi wire. Instrument with greater bending flexibility may generate less unwanted lateral forces in curved canals during root canal preparation.

Although V2H and PTN have variable tapers, this feature did not increase their flexibility in this study. PTN instruments with variable taper were found to be less flexible than the NRT, which has a constant taper. However, additional experiments are required with NiTi rotary files that have identical alloy and cross sections to be able to adequately compare the influence of constant versus variable taper. The present study measured torsional fatigue resistance using a torque-controlled endodontic motor, to mimic clinical condition. Maximum degree of rotation cannot be obtained by this method, and further study on the torsional fracture resistance test according to ISO 3630-1 is needed.

## 5. Conclusions

NiTi rotary instrument manufactured from CM wire by proprietary thermomechanical procedures had greater bending flexibility and torsional fatigue resistance than conventional and M-wire NiTi instruments.

## Figures and Tables

**Table 1:** Results of torsional fatigue resistance tests (*n* = 10/group).

Group	Minimum	Maximum	Mean (SD)^*∗*^
OS	1	1	1 (0)^a^
PTN	1	2	1.4 (0.55)^a,b^
NRT	1	8	3.6 (2.88)^b^
V2H	79	140	114.4 (22.22)^c^

OS, One Shape; PTN, ProTaper NEXT; V2H, V taper 2H. ^*∗*^Groups with different superscript letters indicate a statistically significant difference.

**Table 2:** Cross-sectional area and inner core area at 3 mm cross sections from tip of the tested instruments.

Group	Cross-sectional area (*μ*m^2^)	Inner core area (*μ*m^2^)
OS	90,697	55,992
PTN	101,255	54,321
V2H	110,295	64,774
NRT	135,957	83,905

OS, One Shape; PTN, ProTaper NEXT; V2H, V taper 2H.

## Data Availability

The experimental data used to support the findings of this study are available from the corresponding author upon request.
